# Fancd2 in vivo interaction network reveals a non-canonical role in mitochondrial function

**DOI:** 10.1038/srep45626

**Published:** 2017-04-05

**Authors:** Tingting Zhang, Wei Du, Andrew F. Wilson, Satoshi H. Namekawa, Paul R. Andreassen, Amom Ruhikanta Meetei, Qishen Pang

**Affiliations:** 1 Division of Experimental Hematology and Cancer Biology, Cincinnati Children’s Hospital Medical Center, Cincinnati, OH, USA; 2 Zhejiang Provincial Key Laboratory of Experimental Animal Research, Zhejiang Academy of Medical Sciences, Hangzhou, Zhejiang,310013, China; 3 Division of Reproductive Sciences, Cincinnati Children’s Hospital Medical Center, Cincinnati, OH, USA.

## Abstract

Fancd2 is a component of the Fanconi anemia (FA) DNA repair pathway, which is frequently found defective in human cancers. The full repertoire of Fancd2 functions in normal development and tumorigenesis remains to be determined. Here we developed a Flag- and hemagglutinin-tagged Fancd2 knock-in mouse strain that allowed a high throughput mass spectrometry approach to search for Fancd2-binding proteins in different mouse organs. In addition to DNA repair partners, we observed that many Fancd2-interacting proteins are mitochondrion-specific. Fancd2 localizes in the mitochondrion and associates with the nucleoid complex components Atad3 and Tufm. The Atad3-Tufm complex is disrupted in *Fancd2*−/− mice and those deficient for the FA core component *Fanca*. Fancd2 mitochondrial localization requires Atad3. Collectively, these findings provide evidence for Fancd2 as a crucial regulator of mitochondrion biosynthesis, and of a molecular link between FA and mitochondrial homeostasis.

Fanconi anemia (FA) is a genetic disorder associated with congenital developmental defects, bone marrow failure and predisposition to cancers, particularly acute myelogenous leukemia^1^,^2^,^3^. In recent decades, the function of FA proteins has been extensively studied and very well documented. As a genetically heterogeneous disease, more than a dozen FA and FA-associated proteins (FANCA, −B, −C, −E, −F, −G, −L, −M, FAAP100, FAAP24, FAAP20, HES1, MHF1, and MHF2) form a core complex^4^,^5^. In response to DNA damage or DNA replication stress, this multimeric FA core complex monoubiquitinates two downstream FA proteins, FANCD2 and FANCI, which recruit other components of the FA/BRCA pathway to damaged DNA loci and consequently affect DNA replication, cell cycle control and DNA damage repair processes^4^,^5^,^6^,^7^,^8^.

While many studies point to an essential role for the FA pathway in DNA damage repair and genome maintenance, emerging evidence suggests that FA deficiency causes mitochondrion dysfunction, which may play roles in the pathogenesis of bone marrow failure and leukemia progression in FA. For example, several studies show that FA cells exhibit abnormalities in mitochondrial metabolism, Ca_2_^+^ homeostasis, and gene expression^9^,^10^,^11^. Additional studies revealed mitochondrion defects in FA patients and mice^12^,^13^,^14^,^15^. At the molecular level, it has been shown that FA deficiency causes altered mitochondrial morphology and mitochondrial complex defect, and decreased mitochondrial membrane potential and ATP production^11^,^16^,^17^. More recently, it has been reported that some of the FA proteins are required for removal of damaged mitochondria and reduction of mitochondrial reactive oxygen species (ROS)^18^. Furthermore, the utility of antioxidants shows *in vivo* protective effects against the onset of malignancies and bone marrow failure in FA knockout mice^19^,^20^. Although these studies have indicated a correlation between mitochondrion defects and FA deficiency, direct evidence for a mechanistic link is missing.

In this study, we developed a *Fancd2* knock-in mouse model that enabled the identification of a non-canonical function of the FA pathway in mitochondrion biosynthesis. We further demonstrated that Fancd2 localizes in the mitochondrion and associates with the nucleoid complex components Atad3 and Tufm, thus providing a molecular link between FA and mitochondrial homeostasis.

## Results

### Generation and analysis of 3XFLAG/HA-tagged *Fancd2* knock-in mice

**Figure 1:**
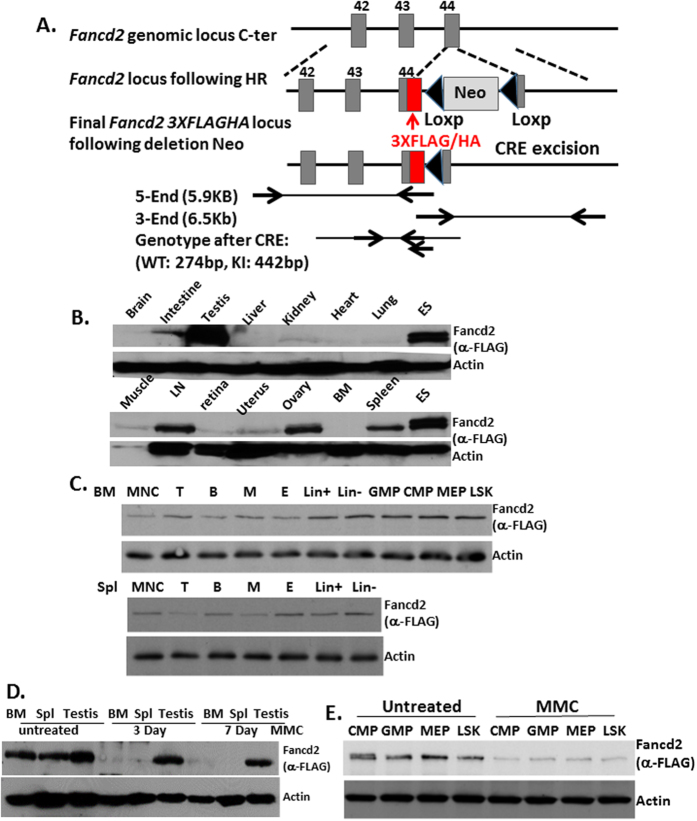
Generation of 3XFLAG/HA-tagged *Fancd2* knock-in mice. (**A**) Schematic diagram of *Fancd2*^*3XFLAG*/*HA*^ (*Fancd2-KI*) mouse knock-in strategy. *Fancd2* genomic locus, *Fancd2* locus following homologous recombination (targeted DNA) and the final *Fancd2*^*3XFLAG*/*HA*^ locus following excision of the *loxP*-flanked *Neomycin resistance* gene (Neo) is illustrated. *Fancd2* exons are shown as gray boxes and are numbered. FLAG/HA (red box) tags are also indicated. (**B**) Tissues isolated from *Fancd2-KI* mice were analyzed by immunoblotting with the M2 anti-FLAG antibody. Fancd2 is highly expressed in ES cells, testes, spleen, lymph nodes and ovaries. (**C**) Bone marrow and spleen mononuclear cells isolated from *Fancd2-KI* mice were sorted and subjected to western blot. Fancd2 is highly expressed in B, erythroid, Lin^−^, MPP populations and relatively low in T, myeloid, Lin^+^ cells. (**D**) Fancd2 expression in bone marrow, spleen and testis is dramatically reduced after a single dose (3 mg/kg) of MMC IP injection in mice. (**E**) Fancd2 expression was analyzed in sorted population cells in bone marrow after MMC treatment. Full-length blot is presented in Supplementary Fig. 4. The data represent a summary of more than three mice of each genotype from two independent experiments.

To study the *in vivo* function of the FA pathway, we generated a *Fancd2* knock-in mouse model, in which a duel tandem (3XFLAG and HA) tag was inserted at the C-terminus of the endogenous *Fancd2* locus (Fig. 1A; Supplemental Fig. 1A,B). We confirmed that the tagged Fancd2 protein was expressed and could be pulled down by FLAG and HA antibodies using 2-step immunoprecipitation in *Fancd2*^*KI*^ ES cells (Supplemental Fig. 1C). In addition, immunoblotting of mouse embryonic fibroblast (MEF) cells from wild-type (WT) and *Fancd2*^*KI*/*KI*^ mice with anti-FLAG and anti-Fancd2 antibodies demonstrated that the tagged Fancd2 and the WT endogenous Fancd2 proteins were expressed at roughly the same levels (Supplemental Fig. 1D), indicating that the FLAG-HA tag does not affect the expression of the Fancd2 protein. Furthermore, treatment of *Fancd2*^*KI*^ ES cells showed that both mitomycin C (MMC) and hydrourea (HU) induced monoubiquitination of the tagged Fancd2 protein (Supplemental Fig. 1E). Immunofluorescence microscopy for *Fancd2*^*KI*/*KI*^ MEF cells with an anti-HA antibody showed that tagged Fancd2 retained the ability to form DNA damage foci in nuclei after MMC treatment (Supplemental Fig. 1F). These results indicate that the tagged Fancd2 protein retains its functionality in the context of the DNA damage response at the cellular level.

To examine the function of the tagged *Fancd2* alleles *in vivo*, we subjected *Fancd2*^*KI*/*KI*^ animals to MMC injection. Unlike *Fancd2-KO (Fancd2*^−/−^) mice, which showed hypersensitivity to MMC and died within 10 days after injection, homozygous *Fancd2*^*KI*/*KI*^ mice were resistant to MMC (Supplemental Fig. 2A). Histopathological analysis showed that the *Fancd2*^*KI*/*KI*^ mice had normal testes and ovaries compared to *Fancd2*^−/−^ mice, characteristic of severe gonadal defects (Supplemental Fig. 2B)^2^. In the context of hematopoiesis, the colony-forming activity of *Fancd2*^*KI*/*KI*^ bone marrow progenitor cells was not affected by MMC at a dose that significantly inhibited the proliferation of *Fancd2*^−/−^ progenitor cells (Supplemental Fig. 2C). We also examined primary MEF cells derived from the homozygous *Fancd2*^*KI*/*KI*^ mice for other two FA cellular hallmarks: MMC-induced cell death and G_2_/M arrest^21^,^22^. Not surprisingly, unlike the *Fanca*^−/−^ or *Fancd2*^−/−^ cells, the *Fancd2*^*KI*/*KI*^ cells were completely resistant to MMC-induced cell death (Supplemental Fig. 2D and G)_2_/M arrest (Supplemental Fig. 2E). Taken together, the *Fancd2*^*KI*/*KI*^ mice display normal development and the tagged Fancd2 protein retains full functionality in the DNA damage response.

### Tissue-specific expression of Fancd2 in mice

We next determined the expression of the Fancd2 protein in different tissues of *Fancd2*^*KI*/*KI*^ mice. We found that Fancd2 was highly expressed in the testes, which is consistent with previously reported data^23^. We also observed high levels of Fancd2 expression in ES cells, lymph node, spleen and ovary tissue (Fig. 1B). Since bone marrow failure and later myeloid malignancies are the major symptoms in FA patients^1^,^2^,^3^, we analyzed Fancd2 expression in different hematopoietic lineages. Fancd2 was found highly expressed in B (B220^+^), erythroid (CD71^+^Ter119^+^), lineage-negative (Lin^−^), committed progenitors and Lin^−^Sca1^+^c-Kit^+^ (LSK) cell populations, and was relatively low in T (CD3e^+^), myeloid (Gr-1^+^CD11b^+^), Lin^+^ cell populations (Fig. 1C). Interestingly, little ubiquitinated (Ub) form of Fancd2 was detected in the fresh tissues or cells, unlike the obvious Ub-Fancd2 in ES cell lines (Fig. 1B).

Since hypersensitivity to MMC-induced DNA damage is the cellular hallmark of FA cells, we examined whether MMC treatment would alter the level of Fancd2 protein *in vivo*. We found that the levels of the Fancd2 protein were dramatically reduced in bone marrow, spleen, testis, committed progenitors and LSK cells after a single dose (3 mg/kg body weight) of MMC injection (Fig. 1D,E). Unexpectedly, MMC treatment did not induce robust Fancd2 monoubiquitination in the testis (Fig. 1D).

### Proteomic analysis of Fancd2-associated proteins

**Figure 2:**
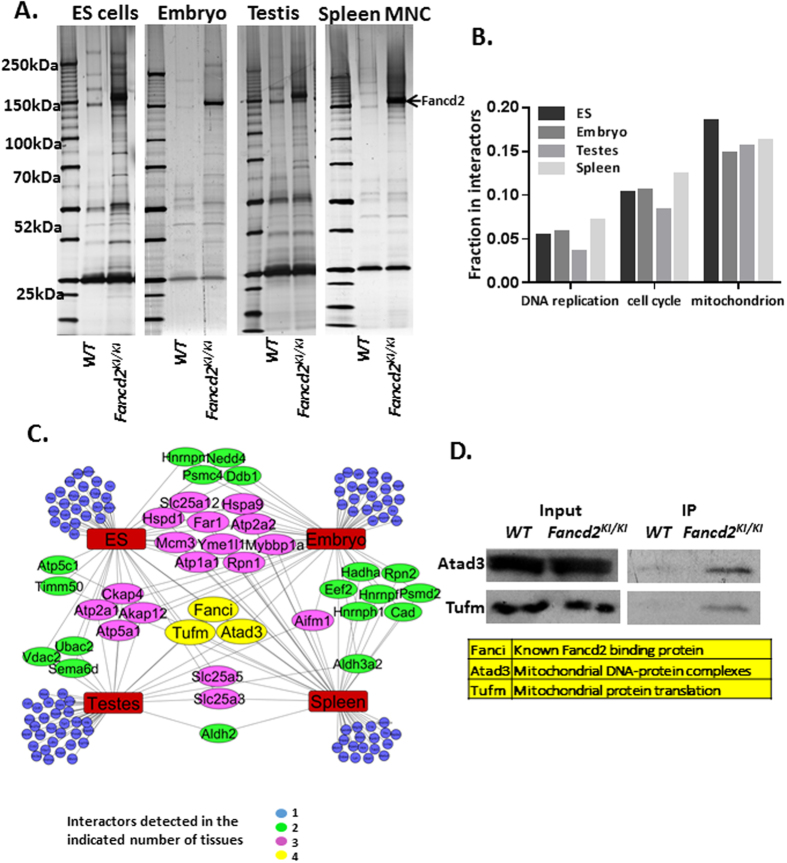
Proteomic analysis of Fancd2-associated proteins. (**A**) Silver stained gels with Fancd2 containing complexes purified from indicated tissues (Fancd2-IP). WT: Mock-IP, mock purification from organs of wild-type mice. Bands corresponding to Fancd2 are marked by an arrow. (**B**) Fractions of proteins, among Fancd2 interactors, classified to indicate Gene Ontology categories show biological process/molecular function enrichment. (**C**) Diagram depicting Fancd2 interactors, grouped by tissue in which they were detected. Each color corresponds to number of occurrences across all four tissues. Blue nodes, only in one tissue, Green nodes, common to two tissues, Purple nodes, common to 3 tissues, Yellow nodes, common to all 4 tissues. (**D**) Interaction of endogenous with Fancd2 to Atad3 and Tufm, detected by anti-FLAG immunoprecipitation and anti-Atad3 and Tufm immunoblotting in spleen MNCs.

To identify Fancd2 interactors *in vivo*, we isolated Fancd2-containing complexes from ES cells, E11.5 mouse embryos, testes and spleen mononuclear cells (Fig. 2A), as these compartments express high levels of Fancd2 (Fig. 1B). The identities of Fancd2-associated proteins were determined by liquid chromatography and tandem mass spectrometry (LC–MS/MS; Supplementary Table 1). We validated the association of Fancd2 with some of the interactors using anti-Flag pull-down. Among them, we detected known Fancd2-binding partners, including Fanci, Msh2 and DDB1 (Supplemental Fig. 3). Screening the interactors for ontology categories showed enrichment for cell cycle (P < 0.001) and DNA replication (P < 0.001) proteins. Unexpectedly, proteins involved in mitochondrion homeostasis were enriched the most (P < 0.001) (Fig. 2B). The Fancd2 interaction network reveals different patterns of Fancd2-associated polypeptides in these four compartments (Fig. 2C), suggesting the presence of developmental and tissue-specific differences in the repertoire of Fancd2 interactors. Significantly, we observed three proteins, Fanci, Atad3, and Tufm that interacted with Fancd2 in all four tissues (Fig. 2C). We confirmed the interaction of Fancd2 with Atad3 and Tufm in the spleen (Fig. 2D). Fanci is a well-known Fancd2-binding partner^7^; whereas Atad3 (component of the mitochondrial nucleoid complex) and Tufm (component of mitochondrial translation machinery) are essential for mitochondrion biosynthesis^24^,^25^,^26^,^27^.

### Loss of Fancd2 or Fanca causes dysregulation of mitochondrion genes

**Figure 3:**
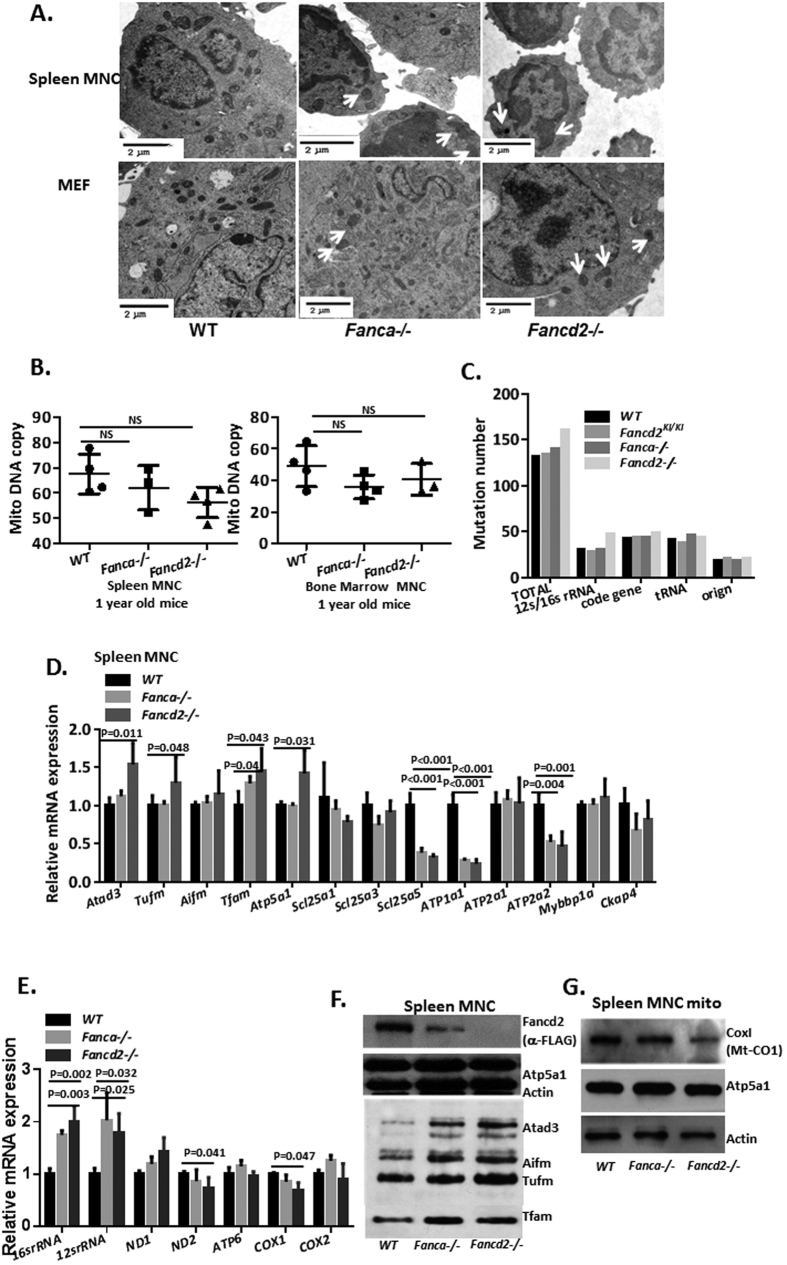
Dysregulation of mitochondrial genes in the absence of Fancd2 or Fanca. (**A**) Transmission electron microscopy of spleen MNCs and MEFs from WT, *Fanca*−/− *and Fancd2*−/− mice. Swollen mitochondria with disorganized cristae are indicated with a white arrow. (**B**) The mtDNA copy number in bone marrow MNCs and spleen MNCs was analyzed by QPCR using primers against mtDNA and nuclear DNA (3 mice for each genotype). No significant difference was observed between the one-year old WT, *Fanca*−/− and *Fancd2*−/− mice with Student’s t-test analysis. (**C**) To analyze mtDNA integrity in FA mice, 16 kb mtDNA of 2 month old mice spleen MNCs was amplified. The PCR product was subjected to small genome sequencing. The *Fancd2*−/− mtDNA shows slightly higher mutation rates compared to *WT, Fancd2*^*KI*/*KI*^ and *Fanca*−/− mice, especially in the 16 s/12 s rRNA region. (**D**) Quantitative PCR analysis shows up-regulation of genes associated with mitochondrial biogenesis (Atad3, Tufm, Tfam) and down-regulation of genes involved in mitochondrial oxidative phosphorylation (Atp1a1, Atp2a2, Scl25a5) in *Fanca*−/− and *Fancd2*−/− spleen MNCs compared to WT spleen MNC cells. The data represent a summary of more than three mice of each genotype from two independent experiments. The P values indicated were analyzed using Student’s t-test analysis. (**E**) For mtDNA-encoded genes, quantitative PCR analysis shows up-regulation of the 16 s rRNA and 12 s rRNA and down-regulation of Cox-1 and Nd2 expression in the *Fanca*−/− and *Fancd2*−/− spleen MNCs comparing to WT cells. (**F**) Western blot shows increased expression of Atad3, Tufm, Aifm in the mitochondrion fraction from *Fanca*−/− and *Fancd2*−/− spleen MNCs. (**G**) Western blot shows decreased Cox-1 expression in the mitochondrion fractions of *Fanca*−/− and *Fancd2*−/− spleen MNCs. Full-length blot is presented in Supplementary Fig. 5.

Since FA proteins have been shown to regulate oxidative stress and mitochondrion respiration complex enzymes^9^,^12^,^13^,^16^,^17^,^18^, and since mitochondrial Fancd2-interactors show the highest enrichment score in our proteomic analysis (Fig. 2 above), we decided to study the link between FA and the mitochondrion further. We first examined whether FA deficiency affected mitochondrion morphology. Transmission electron microscopy analysis revealed swollen mitochondria with disorganized cristae in spleen MNCs and MEFs of *Fanca*−/− and *Fancd2*−/− mice (Fig. 3A).

Since the FA pathway is essential for the response to replicative stress and repair of DSBs generated during DNA replication, we suspected that the FA proteins were directly involved in mtDNA replication and repair. To our surprise, the mtDNA copy number in the bone marrow and spleen MNCs showed no significant difference between the one year old WT, *Fanca*−/− and *Fancd2*−/− mice (Fig. 3B). To determine if mtDNA integrity was impaired in FA-deficient mice, we performed mtDNA sequencing and found no significant difference in mutation rates between WT and *Fanca*−/− or *Fancd2*−/− mtDNA of spleen MNCs (Fig. 3C). These results indicate that loss of Fanca or Fancd2 in adult mice has no effect on mtDNA copy number or the mutation frequency, suggesting that mtDNA variance was likely not the cause of the mitochondrion defect in FA.

To search for clues about Fancd2 function in mitochondria, we first analyzed nuclear- and mtDNA-encoded gene expression at the mRNA and protein levels. Quantitative PCR analysis of nuclear genes functioning in the mitochondrion showed specific up-regulation of genes associated with mitochondrial biogenesis (Atad3, Tufm, Tfam) and down-regulation of genes involved in oxidative phosphorylation (Atp1a1, Atp2a2, Scl25a5) in *Fanca*−/− and *Fancd2*−/− spleen MNCs comparing to WT cells (Fig. 3D). For mtDNA-encoded genes, up regulation of 16 s rRNA and 12 s rRNA and down regulation of ND2 and CoxI mRNA were detected in *Fanca*−/− and *Fancd2*−/− cells (Fig. 3E). Immunoblotting of mitochondrial proteins showed increased expression of nuclear-coded proteins Atad3, Tufm, Aifm and decreased mtDNA-coded CoxI in the spleen MNCs of *Fanca*−/− and *Fancd2*−/− mice (Fig. 3F,G). Collectively, these results indicate that loss of Fanca or Fancd2 leads to an alteration in mtDNA gene expression.

### Fancd2 is required for the stability of the mitochondrion nucleoid complex

**Figure 4:**
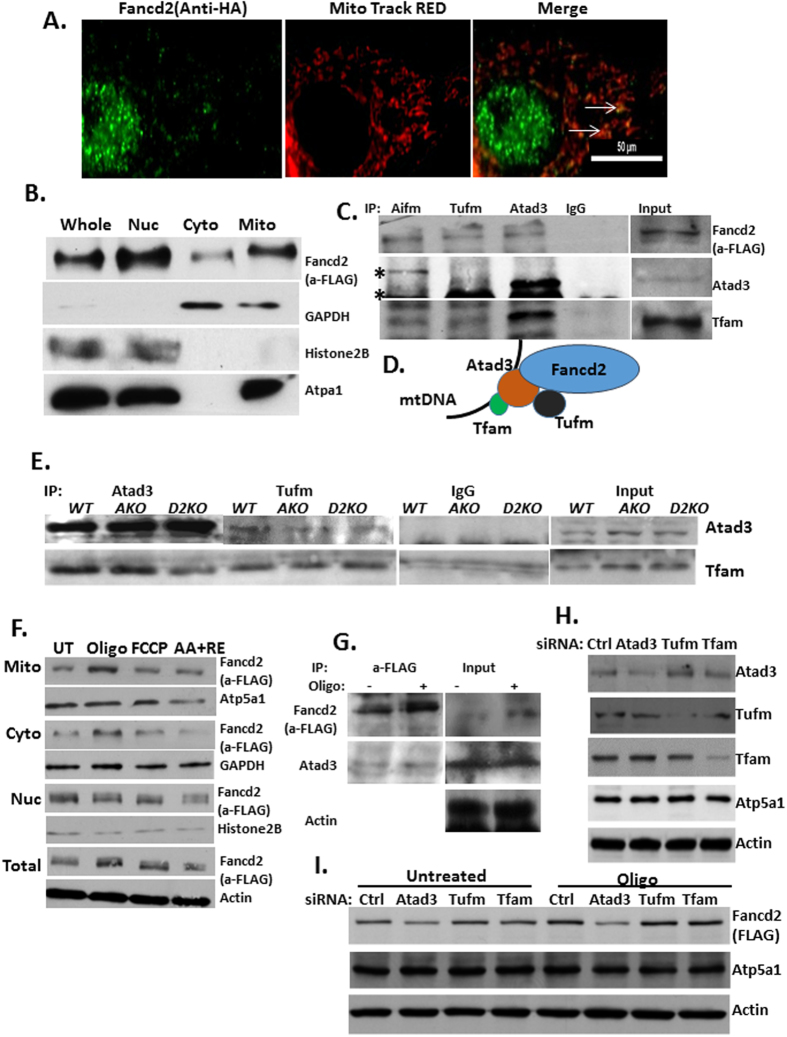
Fancd2 is a component of the mitochondrial nucleoid complex. (**A**) Fancd2 is localized in mitochondria. Anti-HA antibody and FITC-labeled secondary antibody were used to detect tagged-Fancd2 protein in *Fancd2-KI* MEFs. Mito tracker RED was used to label mitochondria. (**B**) Western analysis of the FLAG-tagged Fancd2 protein in MEF cells of *Fancd2-KI* mice in whole cell lysates (Whole), nuclear (Nuc), cytoplasmic (Cyto) and mitochondrial (Mito) fractions. Anti-FLAG was used to probe Fancd2. GAPDH, ATP5A1, and Histone2B were probed as the markers for cytoplasmic, mitochondrial and nuclear controls, respectively. A significant fraction of the Fancd2 protein is localized in the mitochondrion. (**C**) Fancd2 co-immunoprecipitates with Atad3, Tufm and Aifm. Aifm, Atad3 and Tufm immunoprecipitation were performed on *Fancd2*^*KI*/*KI*^ spleen MNCs. Nonspecific band is indicated by an asterisk. (**D**) A model of the Fanc2/Atad3/Tufm complex. (**E**) Effect of FA deficiency on the Atad3/Tufm/Tfam complex. Atad3 and Tufm immunoprecipitation in *Fancd2*^*KI*/*KI*^, *Fanca*−/− *Fancd2*^*KI*/*KI*^ and *Fancd2*−/− spleen MNCs. (**F**) Fancd2 expression responds to mitochondrial stress. *Fancd2*^*KI*/*KI*^ MEFs were treated with different mitochondrion respiration complex inhibitors: 4 uM oligomycin, 4 uM FCCP, or 2 uM antimycin A and 2 uM rotenone for 16 hr. Immunoblot of cell fraction lysates shows an increase in the levels of Fancd2 in the whole cell, mitochondrion and cytosol fraction, but not in the nuclear fractions. (**G**) Immunoblot showing that more Atad3 was co-immunoprecipitated with Fancd2 in oligomycin treated *Fancd2*^*KI*/*KI*^ MEFs. (**H**) Immunoblot of *Fancd2*^*KI*/*KI*^ MEFs lysates shows siRNA knockdown of control, Atad3, Tufm, and Tfam. (**I**) Immunoblot shows reduced Fancd2 in the mitochondrial fraction after Atad3 knockdown with or without oligomycin treatment. Full-length blot is presented in Supplementary Fig. 6.

We then turned our attention to the two mitochondrial Fancd2-interactors, Atad3 and Tufm, which interacted with Fancd2 in all four tissues (Fig. 2C,D). Both Atad3 and Tufm are among the most frequently identified components of the mitochondrion nucleoid complex^24^,^25^,^26^,^27^, which is essential for mitochondrion biosynthesis. In addition, Atad3 and Tufm have been reported to be required for the mitochondrial transcription and translation process^24^,^25^,^26^,^27^,^28^. Since Atad3 and Tufm are localized exclusively in mitochondria and are present in the mitochondrial nucleoprotein complex^25^, we postulated that Fancd2 might play a role in the mtDNA transcription and translation process, probably by stabilizing the Atad3/Tufm/Tfam nucleoid complex. We first determined whether Fancd2 was localized to the mitochondria. Immunofluorescence staining of tagged-Fancd2 in *Fancd2*^*KI*/*KI*^ MEFs with an anti-HA antibody showed that Fancd2 was predominantly localized in the nucleus with a small fraction found in the cytosol (Fig. 4A). Cytoplasmic Fancd2 exhibited co-localization with the mitochondrial maker Mito tracker RED (Fig. 4A), suggesting that Fancd2 is also localized to the mitochondria. To confirm this finding, we performed immunoblotting on fractionated lysates of *Fancd2*^*KI*/*KI*^ MEFs cells. The mitochondrial, cytosol, and nuclear fractions were marked by Atp5a1, GAPDH and Histone2B, respectively. Consistent with the immunostaining result, the Fancd2 protein was detected in both the nuclear and cytosol fractions (Fig. 4B). Importantly, a significant amount of Fancd2 was co-fractionated with Atp5a1 in the mitochondria fraction, adding further support to mitochondrial localization of Fancd2.

We next determined whether Fancd2 was an integral component of the mitochondrion nucleoid complex *in vivo*. To this end, we first performed immunoprecipitation experiments using antibodies specific for Atad3, Tufm, and Aifm (Apoptosis-Inducing Factor, Mitochondrion-Associated), which is a Fancd2 interactor found in testes, spleen and embryos (Fig. 2C,D). Fancd2 co-immunoprecipitated with Atad3, Tufm and Aifm in the lysates of *Fancd2*^*KI*/*KI*^ spleen MNCs (Fig. 4C). In the meantime, Tufm associated with Atad3, which co-immunoprecipitated with Tfam (Transcription Factor A, Mitochondrial) (Fig. 4C), another component of the mitochondrion nucleoid complex^25^. Collectively, these results suggest that Fancd2 is part of the mitochondrion nucleoid complex, probably through interacting with Atad3 and Tufm (Fig. 4D).

We then asked whether loss of FA proteins would affect the Fancd2/Atad3/Tufm complex by carrying out co-immunoprecipitation experiments using *Fancd2*^*KI*/*KI*^, *Fanca*−/−*Fancd2*^*KI*/*KI*^ and *Fancd2*−/− spleen MNCs cells. We observed that the Atad3/Tufm interaction was reduced in the absence of Fanca and Fancd2 (Fig. 4E). Altogether, loss of Fanca or Fancd2 disrupted Fancd2/Atad3/Tufm mitochondrion nucleoid complex, which likely contributes to the mtDNA transcription and translation defect in FA cells.

### Atad3 mediates mitochondrial import of Fancd2

Since the expression of numerous genes encoding mitochondrial proteins, including Atad3, is induced by mitochondrial dysfunction leading to a protective response^29^,^30^, we examined next whether the expression of Fancd2, as an Atad3-interacting partner, also responded to mitochondrial stress. We treated *Fancd2*^*KI*/*KI*^ MEFs with different mitochondrion respiration complex inhibitors oligomycin (Complex V inhibitor), FCCP (Uncoupling agents), or antimycin A (Complex III inhibitor) combined with rotenone (complex I inhibitor). We observed that the level of Fancd2 was significantly increased 16hr after oligomycin treatment, in the mitochondrion and cytosol fractions but not in the nuclear fraction (Fig. 4F). In addition, more Atad3 was co-immunoprecipitated with Fancd2 in oligomycin treated MEFs (Fig. 4G). These results indicate that Fancd2 is involved in the response of cells to mitochondrial stress.

Atad3 has been found anchored in the inner mitochondrial membrane and to deliver substrate into mitochondria^31^. This prompted us to ask whether Atad3 mediated Fancd2 import to mitochondria. *Fancd2*^*KI*/*KI*^ MEFs were transfected with siRNA targeting Atad3, Tufm or Tfam, and then analyzed for mitochondrial Fancd2. We obtained significant knockdown (>80%) for these proteins in the mitochondrion (Fig. 4H). Knocking down Atad3 but not Tufm or Tfam ablated Fancd2 in both resting (untreated) and stressed (oligomycin-treated) mitochondria (Fig. 4I). Thus, Atad3 is indispensable for the localization and importation of Fancd2 to the mitochondrion with or without stress.

## Discussion

Most studies on FA cellular and molecular mechanisms have been conducted in cultured cell lines *in vitro*. For *in vivo* studies, several FA knockout mice have been reported, to date, with only a few phenotypic features recapitulating FA patients, including hypersensitivity to DNA-crosslinking agents, abnormal gonads and stressed hematopoiesis^32^,^33^. Studies on FA mice or human patient-derived primary cells identified additional phenotypes including apoptotic response deregulation, oxidative stress hypersensitivity, and mitochondrion deficiency, which all appear to fall outside of DNA damage repair^9^,^10^,^11^,^12^,^13^,^14^,^15^,^16^,^17^,^18^,^19^,^20^. Using an innovative *Fancd2* knock-in mouse model, the recent study shows that most Fancd2-binding proteins do not have a recognized function in DNA damage response and repair. Particularly, many Fancd2-interactors are mitochondrion-specific. We also show that Fancd2 is localized in the mitochondrion and associates with the nucleoid complex components Atad3, Tufm and Tfam, all of which are required for mitochondrial biosynthesis. Moreover, the Atad3/Tufm/Tfam complex is disrupted in *Fancd2*−/− mice and those deficient for the FA core component *Fanca*. These findings add another facet to the non-repair function of FA proteins.

A major limitation for studying the molecular mechanisms in FA murine models is the lack of antibodies with high-affinity for mouse FA proteins. We generated a 3 × FLAG-HA tagged Fancd2 knock-in mouse model that overcomes this limitation and this has enabled us to identify Fancd2-binding proteins in different organs, thus gaining new insights into FA protein function *in vivo*. We have shown that the 3 × FLAG/HA tagged Fancd2 is essentially wild-type and that the homozygous *Fancd2*^*KI*/*KI*^ mice are indistinguishable from their wild-type counterparts. The 3 tandem FLAG is ideal for detecting Fancd2 in a small amount or for immunoprecipitation. Indeed, this duel tag has enabled us to perform 2 step purification of the Fancd2-containing complex with limited amount of materials from different tissues.

A thorough examination of *Fancd2*^*KI*/*KI*^ mice reveals tissue- and cell type-specific expression patterns that likely correspond to differential Fancd2 function, especially in the reproductive and hematopoietic organs. The general absence of Fancd2 monoubiquitination in the organs of the *Fancd2*^*KI*/*KI*^ mice are particularly striking and suggest that a low level of DNA repair activity of Fancd2 may be sufficient for the maintenance of tissue homeostasis *in vivo*. Conversely, we detected a substantial amount of monoubiquitinated Fancd2 in ES cells and MEFs derived from *Fancd2*^*KI*/*KI*^ embryos, suggesting that environmental exposure during *in vitro* cell culture may have induced Fancd2 monoubiquitination, likely by triggering cellular stress including DNA damage. These findings demonstrate a very different *in vivo* operation of the FA pathway with a majority of the Fancd2 protein exhibiting as non-ubiquitination form, which leads us to propose the existence of ubiquitination-independent functions outside of DNA repair. However, it is also possible that the status of Fancd2 monoubiquitination may reflect cell proliferation or tissue turnover.

Our proteomic study reveals that the most predominant Fancd2 interactors in all four tested tissues belong to those involved in mitochondrial homeostasis, suggesting FA deficiency as a potential contributing factor to mitochondrion deficiency. This intriguingly universal interaction between Fancd2 and mitochondrial proteins also lends support to previous observations that FA proteins regulate oxidative stress response and mitochondrion functions^9^,^12^,^13^,^16^,^17^,^18^. Remarkably, we identified two important mitochondrial proteins, Atad3 and Tufm, with which Fancd2 interacts in all four tissues. Atad3 and Tufm are among the most frequently identified components in the mitochondrion nucleoid complex^27^. Since the mitochondrion nucleoid complex participates in mitochondrial protein synthesis characteristic of uniquely coupled transcription and translation processes^34^,^35^, both Atad3 and Tufm proteins have been demonstrated to be required for mitochondrial transcription and translation processes^24^,^27^. In contrast to the assumption that Fancd2 impacts mtDNA replication and integrity, we found that Fancd2 interferes with mtDNA-encoded gene transcription and translation, by interacting with, and stabilizing, the Atad3/Tufm/Tfam mitochondrial nucleoid complex. Indeed, our results indicate that Atad3 mediates Fancd2 localization and importation to mitochondria. Additionally, the deregulation of nuclear-encoded mitochondrial proteins in the *Fanca*- and *Fancd2*-deficient cells appear to be part of the feedback loop ultimately leading to mitochondrial dysfunction. However, the exact mechanism underling the functional interaction between Fancd2, the Atad3, Tufm, Tfam mitochondrial nucleoid complex and nuclear-coded mitochondrial proteins remains to be further investigated. Also, how Fanci or Fanca regulates Fancd2 in mitochondrial function remains unknown. Nevertheless, these findings shed new light into the potential link between FA and mitochondrial defects.

Further investigation is needed to determine whether the Fancd2-Atad3-Tufm complex formation requires Fancd2 monoubiquitination or an intact FA core complex. Since we do not observe monoubiquitinated Fancd2 in the cytoplasm or mitochondria, we envision another intriguing scenario, where both a non-monoubiquintinated Fancd2 or the Fancd2 protein in cells lacking the key FA core component Fanca could still form complexes with Atad3-Tufm. However, given that mice deficient for several components of the FA core complex (e.g. Fanca, Fancb, Fancc, Fancg) show exactly the same phenotypes as those deleted for *Fancd2*^19^,^20^,^21^,^22^,^23^,^32^,^33^, we also speculate that both Fancd2/Fanci monoubiquitination and FA core complex have a crucial role in Fancd2-Atad3-Tufm complex formation.

In summary, the present study employs an innovative *Fancd2* knock-in mouse model to demonstrate that, in addition to its well-established role in DNA repair, Fancd2 influences gene expression and post-translational function of mitochondrial proteins Atadf3, Tufm, and Tfam.

## Methods

### Generation of 3XFLAG/HA-tagged *Fancd2* knock-in mice

The C-terminally tagged allele (*Fancd2*^*3XFlag*/*HA*^) was generated by inserting sequences encoding 3XFLAG/HA tags immediately upstream of the STOP codon (TGA) within the forty-forth exon of the *Fancd2* gene (Fig. 1A). Fancd2 targeting vectors were constructed by recombineering as previously described^36^. The linearized targeting plasmid was electroporated into 129/B6 F1 hybrid V6.5 embryonic stem cells (Novus biologicals) and DNA from G418-resistant ES cell clones were analyzed. The floxed Neomycin selection marker was then excised by transient expression of Cre recombinase. Positive ES cell lines were then injected into mouse blastocysts. Chimeric animals were obtained and crossed to C57BL/6J mice, and homozygous *Fancd2*^*KI*/*KI*^ mice on a mixed 129S4, C57BL/6J background were generated. Founder animals were also backcrossed to pure C57BL/6J mice for six generations to generate a population of homozygous *Fancd2*^*KI*/*KI*^ mice on the C57BL/6J background.

### Purification of Fancd2-containing complexes, mass spectrometry and proteomic analysis

For one purification round of Fancd2-containing complexes, we pooled around 40 10 cm dishes of cultured ES cells, or 20 11.5D embryos, or 8 adult testes, or 24 adult spleens. The same number of cells or organs from WT mice was used for mock purifications. Organs were lysed using a Homogenizer, on ice, in 3 volumes of ice-chilled Lysis buffer with Roche Complete proteinase inhibitor. Lysates were cleared by centrifugation (40,000 g, 20 min, at 4 °C), and the supernatant was incubated with 100 ul of agarose beads conjugated to anti-FLAG antibody (M2, Sigma) at 4 °C. After a few washing steps with ice-cold lysis buffer, Fancd2, together with the associated proteins was eluted in 400 ul of the lysis buffer containing 200ug/ml FLAG peptide (Sigma). The eluate was incubated with 20 ul of anti-HA beads (Roche) for 4 hrs at 4 °C. After several washes with the lysis buffer, Fancd2 together with the associated proteins was boiled and run on a NuPAGE Bis-Tris gel (Invitrogen). In parallel, we performed identical “mock” purifications from the same number of organs or tumors dissected from wild-type animals (which do not express tagged Fancd2). The final samples were submitted for Liquid chromatography and tandem mass spectrometry (LC-MS/MS) at the Taplin Biological Mass Spectrometry Facility, Harvard Medical School.

All peptides and corresponding spectral count results are shown in Supplementary Table 1. Each protein was evaluated for mock or Fancd2 immunoprecipitated samples. All proteins that were present in mock samples were excluded from further analysis for each tissue. We observed about 200–300 proteins for each tissue. Proteins with more than 1 or 2 peptides were subjected to further study. Toppgene was used for Gene Ontology (GO) categories enrichment analysis. STRING and Cytoscape were used for protein interaction study.

### Mice

*Fanca*+/− and *Fancd2* +/− mice were provided by Dr. Madeleine Carreau (Laval University) and Dr. Markus Grompe (Oregon Health & Sciences University)^33^,^37^, respectively. All animals were sacrificed 2–3 weeks after the last injection for experiments. Mice were maintained on a C57BL/6J background in the animal barrier facility at Cincinnati Children’s Hospital Medical Center. Animals were kept in accordance with the protocol approved by the CCHMC Institutional Animal Care and Use Committees. All animal experiments were performed in accordance with the institutional guidelines and were approved by the Institutional Animal Care and Use Committee of Cincinnati Children’s Hospital Medical Center (IACUC2013-0159).

### Flow cytometry

All antibodies were obtained from eBioscience or BD Pharmingen, unless otherwise indicated. Samples were examined on Fortessa or LSRII flow cytometers, and the cytometry data was analyzed with BD FASC Diva or FlowJo software. For lineage cell analysis: Anti-CD71 and anti-Ter119 for erythroid, anti-Gr1 and anti-CD11b for granulocytic/monocytes, anti-CD45R (B220) for B-lymphoid, and anti-CD3e for T-lymphoid. For quantification of CMP, MEP, GMP, CLP, LK and LSK progenitors, mono-nucleated cell were separated by HISTOPAQUE 1083 (sigma) spin, then labeled with a cocktail of anti-mouse lineage markers (CD3e, B220, Ter119, Mac1, and Gr1), anti-mouse CD117 (cKit), and anti-Ly-6A/E (Sca1), anti-CD34, anti-CD16/32, anti-CD150, anti-CD48 and anti-IL-7Ra. Common myeloid progenitors (CMPs) were defined as Lin-Sca1-cKit + CD34 + CD16/32low, and granulocyte-macrophage progenitors (GMPs) were defined as Lin-Sca1-cKit + CD34 + CD16/32+. Megakaryocyte-erythroid progenitors (MEPs) were defined as Lin-Sca1-cKit + CD34-CD16/32-. Cell sorting was performed with a BD FACSAria II at the Flow Cytometry Core facility in CCHMC.

### Western blotting and immunoprecipitation

The Thermo Scientific Mitochondria Isolation Kit and Nucleus Isolation Kit were used for cell fraction isolation. The extracts were obtained and proteins were solubilized for immunoblotting. The following antibodies were used: HA.11 Clone 16B12 (Covance, MMS-101P), FLAG M2-Peroxidase (HRP) (sigma, M8592), beta-actin (sigma), Anti-ATP5A1 antibody (abcam, ab14748), MYBBP1A Antibody (santa cruz, sc-133800), Anti-PP2A Antibody (Millipore,05-421), Hsp60 (Pierce, MA3-012), Cdk1 Antibody (santa cruz, sc-53219), MSH2 Antibody (Novus, NB100-56428), Anti-Histone H2B antibody (Abcam, ab18977), GAPDH (14C10) (cell signaling, 3683), FANCD2 (novusbio, NB100-182), AIF Antibody (santa cruz, sc-5586), ATAD3A/B/C (santa cruz, sc-292156), EF-Tu (santa cruz, sc-367739), Anti-ALDH2 (sigma, SAB2501484), mtTFA (santa cruz, sc-23588), Anti-MTCO1 antibody (abcam, ab14705). EZview™ Red Anti-HA Affinity Gel (sigma, E6779-1ML), anti-HA beads (Roche), ANTI-FLAG^®^ M2 Affinity Gel (Sigma, A2220-1ML), Protein G Agarose (Roche, 11719416001) were used for IP.

### MtDNA copy number

Total DNA was extracted from cell samples using the DNeasy Blood and Tissue kit (Qiagen, USA). To quantify the mtDNA copy number, real-time PCR was performed using iTaq Universal SYBR Green Supermix (Bio-rad, USA) with a Bio-Rad CFX system. For determination of the amount of nuclear DNA, the *apoB* gene and *B2M* gene were used as a reference. The quantification using the *COXII* or *ND4* region in mtDNA showed similar results. These real-time qPCRs were carried out in quadruplicate for all measurements. All the primers are listed in Supplementary Table S2.

### MtDNA sequencing

The integrity of mtDNA was analyzed by long range PCR, using TaKaRa PrimeSTAR *GXL* DNA polymerase (Qiaqen) to generate an approximately 16 k bp molecule from primers spanning the entire 16 kb mouse mitochondrial genome (forward 5′-CCCAGCTACTACCATCATTCAAGT-3′ and reverse 5′-GAGAGATTTTATGGGTGTAATGCGG-3′). The PCR product was gel purified and submitted to small genome sequencing at the DNA sequencing facility of CCHMC. The NGS result was analyzed by Nextgene software.

### siRNA transfection

siRNA for Tufm (Qiagen, S101458919), Atad3 (Qiagen, S100905723), Tfam (Qiagen, S100183974), and Ubr5 (Sigma, EMU08461) was transfected to the MEFs using Lipofectamine™ RNAiMAX reagent. The effect of targeted siRNA sequences was compared with negative control siRNA (Qiagen).

### Statistical analysis

Student’s t-test and ANOVA test, were performed using Microsoft EXCEL software or Prism 6.0 software (GraphPad Software Inc). Error bars indicate SD. Differences were judged as significant if the P value was <0.05 (*), <0.01 (**) or <0.001 (***).

## Additional Information

**How to cite this article**: Zhang, T. *et al*. Fancd2 in vivo interaction network reveals a non-canonical role in mitochondrial function. *Sci. Rep.* **7**, 45626; doi: 10.1038/srep45626 (2017).

**Publisher's note:** Springer Nature remains neutral with regard to jurisdictional claims in published maps and institutional affiliations.

## Supplementary Material

Supplementary Materials

Supplementary Table 1

Supplementary Table 2
