# Supplementary material for: Fancd2 in vivo interaction network reveals a non-canonical role in mitochondrial function
Source: Sci Rep. 2017 Apr 5;7:45626. doi: 10.1038/srep45626 (PMC5381226; doi:10.1038/srep45626)
Supplement: Supplementary Materials [file srep45626-s1.doc]

**Fancd2 in vivo interaction network reveals a non-canonical role in mitochondrial function**

Tingting Zhang^1, 2^, Wei Du^1^, Andrew F. Wilson^1^, [Satoshi H. Namekawa](http://jcb.rupress.org/search?author1=Satoshi+H.+Namekawa&sortspec=date&submit=Submit)^3^, [Paul R. Andreassen](http://jcb.rupress.org/search?author1=Paul+R.+Andreassen&sortspec=date&submit=Submit)^1^, Amom Ruhikanta Meetei^1,*^, Qishen Pang^1,*^

^1^Division of Experimental Hematology and Cancer Biology, Cincinnati Children's Hospital Medical Center, Cincinnati, OH, ^2^Experimental Animal Research Center, Zhejiang Academy of Medical Sciences,  Hangzhou, Zhejiang,310013, China, ^3^Division of Reproductive Sciences, Cincinnati Children's Hospital Medical Center, Cincinnati, OH

***Address correspondence to:** Qishen Pang, Division of Experimental Hematology and Cancer Biology, Cincinnati Children's Hospital Medical Center, Cincinnati, Ohio 45229. Phone: (513) 636-1152. Fax: (513) 636-2880. E-mail: [Qishen.pang@cchmc.org](mailto:Qishen.pang@cchmc.org), or Amom Ruhikanta Meetei, Division of Experimental Hematology and Cancer Biology, Cincinnati Children's Hospital Medical Center, Cincinnati, Ohio 45229. Phone: (513) 636-1768. Fax: (513) 636-2880. E-mail: Ruhikanta.meetei@cchmc.org.

**Supplementary Materials**

**Supplementary Table 1. Original mass spectrometry results**

**Supplementary Table 2. List of primers used in the experiments**

**Supplementary Figures 1-6**


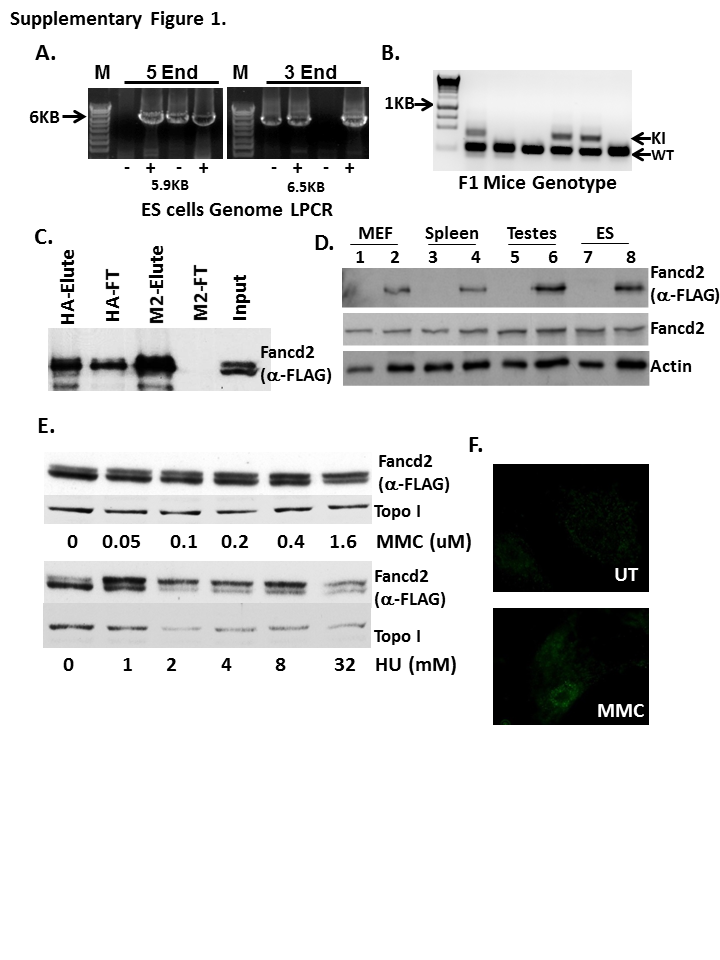


**Supplementary Figure 1. Analysis of 3×FLAG/HA tagged *Fancd2* alleles.** (A) Genotyping by PCR. Two PCR primer sets were used for analyzing the *Fancd2^KI^* allele in positive ES clones. For both 5′ and 3′ sets, one end of the primer sets was designed outside the homology arm and the other end within the *3XFLAG/HA* sequence. The 5′ sets produced a predicted 5.9 kb amplicon from the recombinants. The 3′ sets produced a predicted 6.5 kb amplicon from the recombinants. ES clone with correct 5’ and 3’ PCR product was used for microinjection. (B) Genotyping of the F1 offspring. The internal primer set produced a 274-bp amplicon from the wild-type allele and a 442-bp amplicon from the knock-in allele. (C) 2-step immunoprecipitation was performed on *Fancd2^KI^* ES cells with anti-FLAG and anti-HA antibodies, and the immunoblot was probed with the M2 anti-FLAG antibody. (D) Western blot of the wild-type and *Fancd2^KI/KI^* MEFs with the M2 anti-FLAG and anti-FANCD2 antibody (Novus, NB100-182). Note that the tagged-Fancd2 protein is expressed at roughly the same ratio to the wild-type Fancd2 protein. (E) Western blot of *Fancd2^KI^* ES cells following treatment with serial concentrations of MMC or HU probed with M2 anti-FLAG antibodies. Note that MMC or HU treatment induces monoubiquitination of the tagged-Fancd2 protein. (F) Immunofluorescence staining of *Fancd2^KI^* MEFs with anti-HA antibody. Note that tagged-Fancd2 forms nuclear foci after MMC treatment.

**
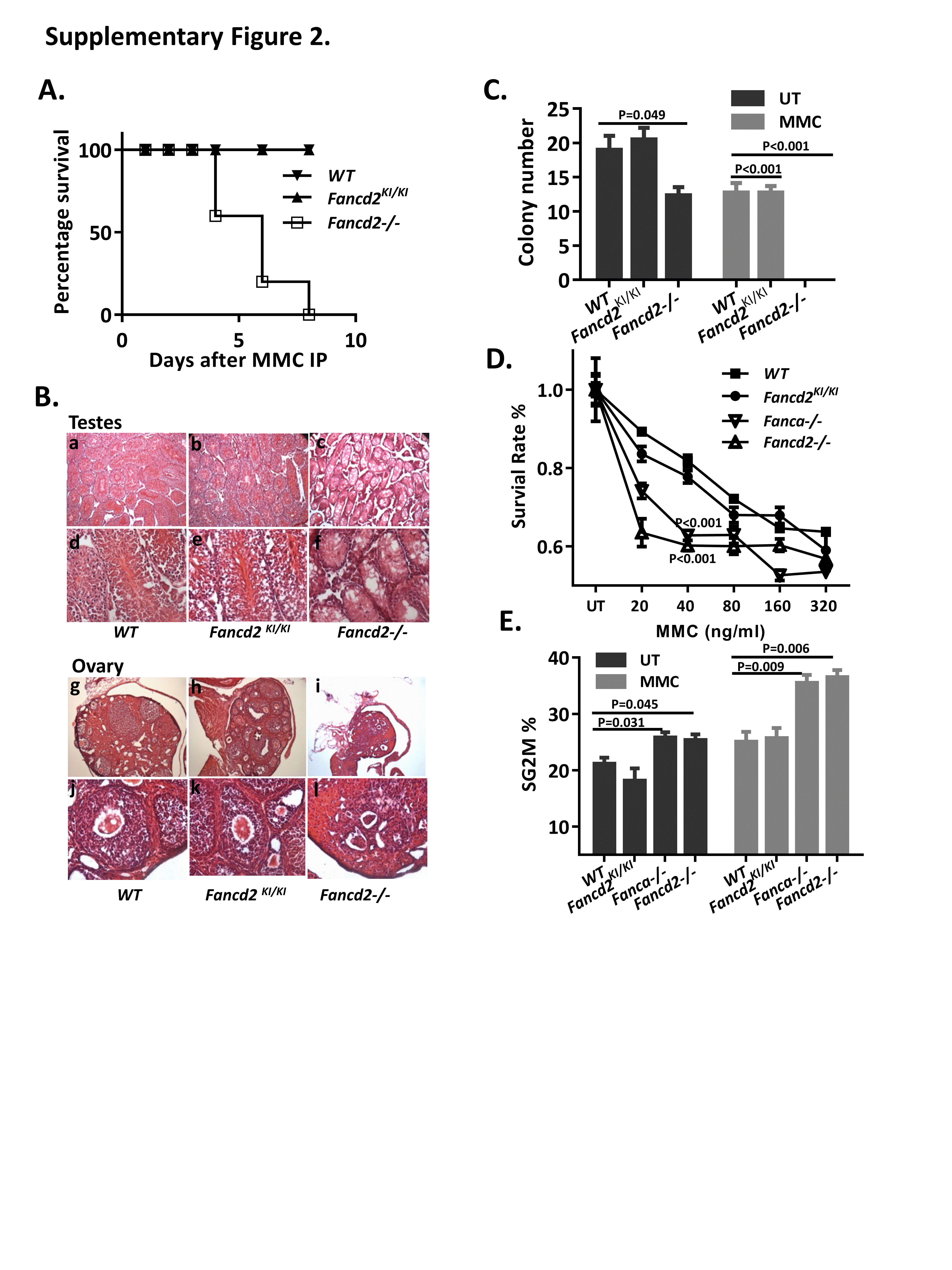
**

**Supplementary Figure 2. Normal development of the *Fancd2^KI/KI^* mice.** (A) *Fancd2-/-* mice, but not WT and *Fancd2^KI/KI^* mice, show hypersensitivity to MMC-induced killing. 6 or more mice were subjected to one dose (3mg/Kg body weight) of MMC IP injection for each group. (B) Gonadal defects in *Fancd2-/-* mice, but not in WT and *Fancd2^KI/KI^* mice. H&E stained sections of testes (a-f ) and ovaries (g-l) from 8-week-old wild type (a,d,g,j) , *Fancd2-/-* (b,e,h,k) and *Fancd2^KI/KI^* (c,f,I,l) mice. Note that the *Fancd2-/-* mice, but not WT and *Fancd2^KI/KI^* mice, exhibit severe organ degeneration ( a,b,c,g,h,I, 50× magnification; d,e,f,j,k,l, 200× magnification). (C) Colony-forming activity of bone marrow progenitor cells from WT, *Fancd2 ^KI/KI^* and *Fancd2-/-* mice. Note that the bone marrow progenitor cells from *Fancd2-/-* mice, but not those of WT and *Fancd2^KI/KI^* mice, show significantly decreased colony-forming ability before and after MMC treatment. Data summarize more than three mice of each genotype from three independent experiments. The P values indicated were obtained using Student’s t-test analysis. (D) *Fancd2-/-* and *Fanca-/-* MEFs are hypersensitive to MMC treatment. MEFs were plated after treatment with or without MMC for 24 hours and colonies were counted after 8 days. Data represent percentage of survival, comparing each dose to untreated cells. Note that the *Fancd2^KI/KI^* MEFs show similar resistance to MMC as WT MEFs. (E) *Fancd2-/-* and *Fanca-/-* MEFs show increased S/G_2_/M phase than wild type and *Fancd2^KI/KI^* MEFs after MMC treatment. The indicated genotypes of MEF cells were stained for PI and Ki67, and analyzed by FACS 24 hours after MMC treatment. Data represent summary of more than three MEFs samples of each genotype from two independent experiments. The P values indicated were obtained using Student’s t-test analysis.

**
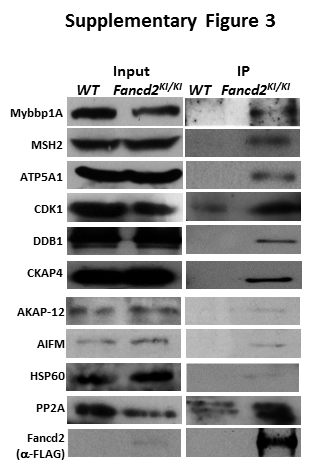
**

**Supplementary Figure 3.** Validation of selected Fancd2-associated proteins by immunoprecipitation with the M2 anti-FLAG antibody.

**
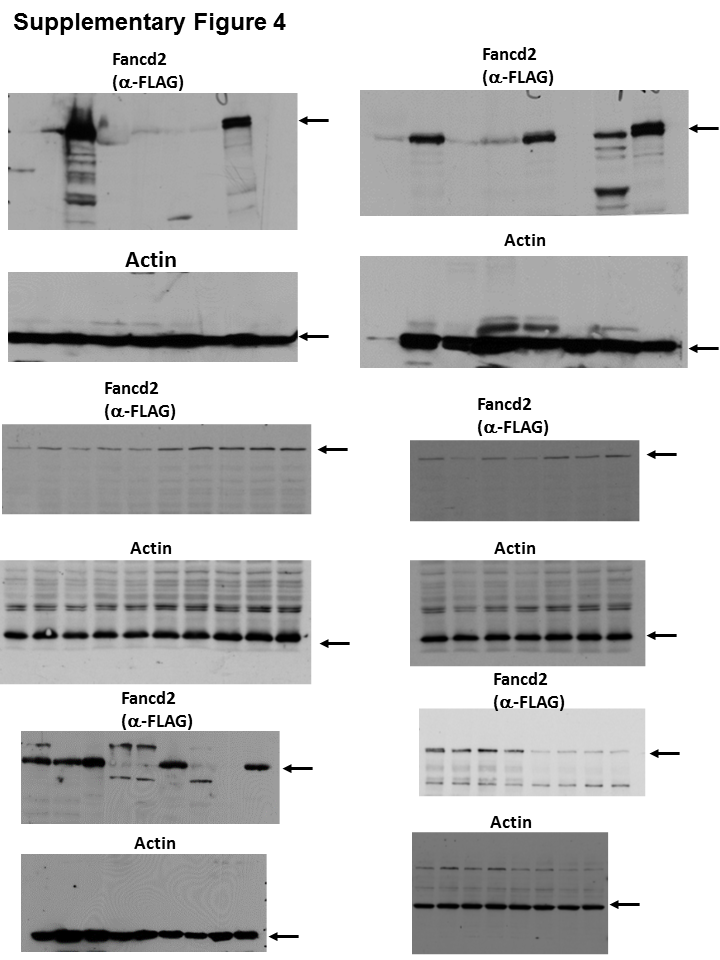
**

**Supplementary Figure 4. Full Blots for Fig. 1.**


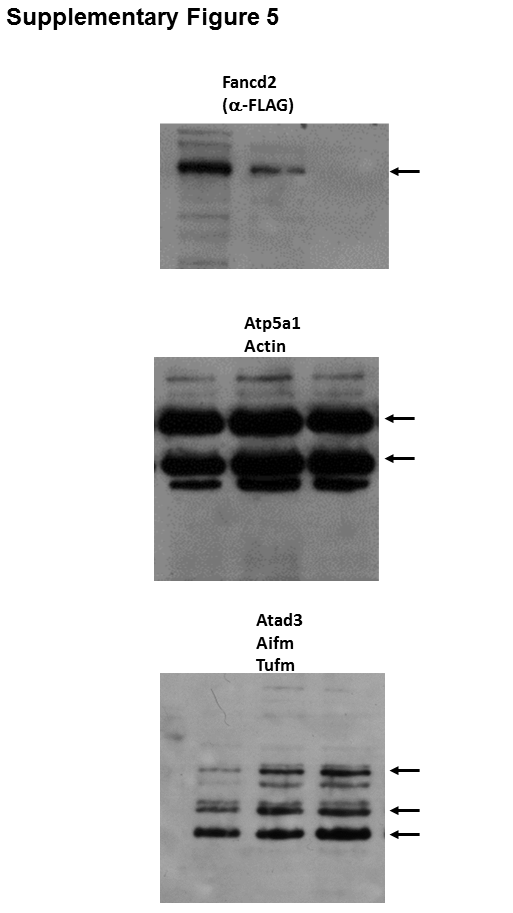


**Supplementary Figure 5. Full Blots for Fig. 3.**


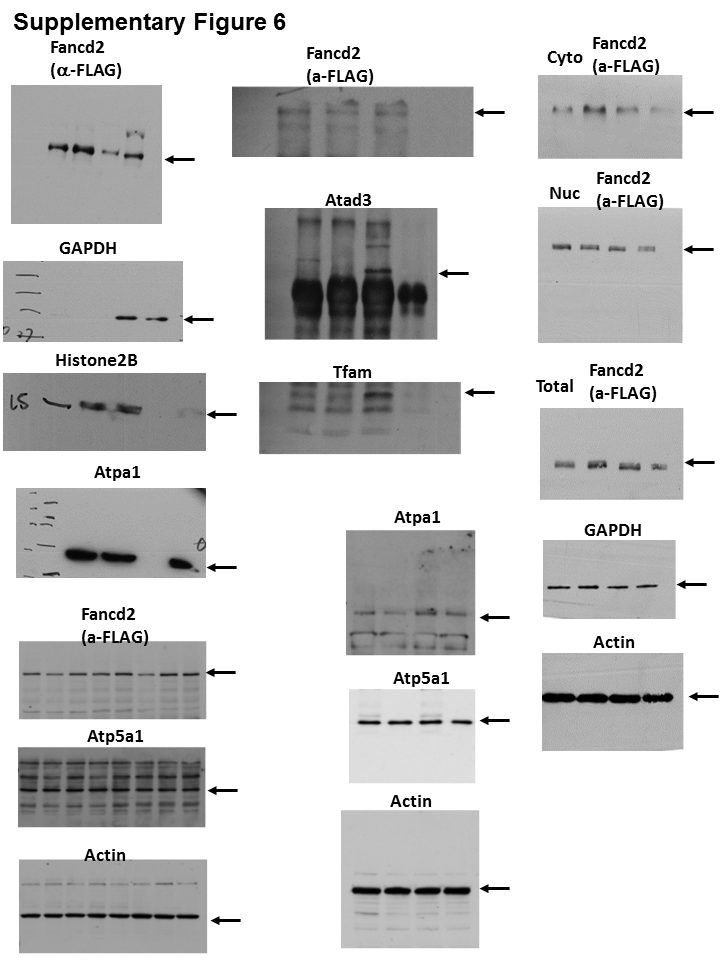


**Supplementary Figure 6. Full Blots for Fig. 4.**
